# Application of a 3D hydrogel-based model to replace use of animals for passaging patient-derived xenografts

**DOI:** 10.1007/s44164-023-00048-x

**Published:** 2023-05-09

**Authors:** Sal Jones, Jennifer C. Ashworth, Marian Meakin, Pamela Collier, Catherine Probert, Alison A. Ritchie, Catherine L. R. Merry, Anna M. Grabowska

**Affiliations:** 1 Ex Vivo Cancer Pharmacology Centre, Translational Medical Sciences, School of Medicine, Biodiscovery Institute, University of Nottingham, Nottingham, UK; 2 Stem Cell Glycobiology Group, Biodiscovery Institute, University of Nottingham, Nottingham, UK; 3 School of Veterinary Medicine & Science, University of Nottingham, Nottingham, UK

**Keywords:** Breast cancer, In vitro model, 3D culture, Tumour microenvironment, Hydrogel, PDX

## Abstract

**Purpose:**

This 3D in vitro cancer model for propagation of patient-derived cells, using a synthetic self-assembling peptide gel, allows the formation of a fully characterised, tailorable tumour microenvironment. Unlike many existing 3D cancer models, the peptide gel is inert, apart from molecules and motifs deliberately added or produced by cells within the model.

**Methods:**

Breast cancer patient-derived xenografts (PDXs) were disaggregated and embedded in a peptide hydrogel. Growth was monitored by microscopic examination and at intervals, cells were extracted from the gels and passaged on into fresh gels. Passaged cells were assessed by qPCR and immunostaining techniques for the retention of characteristic markers.

**Results:**

Breast cancer PDXs were shown to be capable of expansion over four or more passages in the peptide gel. Contaminating mouse cells were found to be rapidly removed by successive passages. The resulting human cells were shown to be compatible with a range of common assays useful for assessing survival, growth and maintenance of heterogeneity.

**Conclusions:**

Based on these findings, the hydrogel has the potential to provide an effective and practical breast cancer model for the passage of PDXs which will have the added benefits of being relatively cheap, fully-defined and free from the use of animals or animal products. Encapsulated cells will require further validation to confirm the maintenance of cell heterogeneity, genotypes and phenotypes across passage, but with further development, including the addition of bespoke cell and matrix components of the tumour microenvironment, there is clear potential to model other cancer types.

**Supplementary Information:**

The online version contains supplementary material available at 10.1007/s44164-023-00048-x.

## Introduction

Close-to-patient cells may be grown in 3D for either expansion or experimentation in both in vivo and in vitro models. Close-to-patient models are those which feature cells expanded directly from patient tissues without growth on plastic or in medium highly enriched for nutrients. The major in vivo technique is patient-derived xenograft (PDX) models, in which human tumours are established and expanded in immunocompromised mouse hosts [1] which results in the sacrifice of a large number of mice for expansion with no direct research output. Organoid models are the most common in vitro approach, in which cells from human tumours are grown in Matrigel with supplementation. In comparison with alternatives such as immortalised cell lines and 2D in vitro experiments, which have otherwise dominated cancer research, both provide a 3D environment and use close-to-patient cells making them more representative of the patient tumour. PDX models additionally are grown in a physiological setting. Thus, these models are increasingly being considered the gold standard for pre-clinical trials of cancer treatments [2–5].

However, both have limitations from both a scientific and animal welfare point of view. Particularly in the context of tumour cell expansion, the PDX model comes with two drawbacks: firstly, the ethical implications of sacrificing many mice without direct research output, and secondly, the influence of signalling from murine non-cancer cells in the tumour. With a growing body of research emphasising the role of the tumour microenvironment (TME) in critical tumour behaviours such as growth rate and treatment responses [6, 7], it is important to consider the effects of non-tumour cells and molecules in a cancer model. In the PDX model, the human tumour stroma, initially transplanted into the mouse alongside the tumour cells, is rapidly replaced by murine counterparts, creating a mismatch between transplanted tumour cells and those in the patients they are intended to represent. The use of immunocompromised mice also means a lack of any immune cell representation. Organoids, while grown in 3D in vitro, also lack stromal cells and require the use of animal-derived basement membrane extract [8, 9]. Since this is an animal-derived product, its use again results in the sacrifice of animals without scientific outputs, and its composition is subject to batch-to-batch variation and does not match that of the extracellular matrix (ECM) in patient tumours which is characteristic of the tumour type and stage [10]. Other limitations are the high costs of these models, putting them beyond the reach of many laboratories.

The ideal would be to create a 3D in vitro expansion model which can be used to passage close-to-patient cells, avoiding the use of a mouse host and Matrigel; such a model would additionally need to allow incorporation of human stromal signals such as relevant ECM components.

We have previously demonstrated the application of a FEFEFKFK peptide gel for modelling both breast cancer and healthy breast tissues [11] and leukaemia [12] in user-defined environments. The gel’s alternating polar (E = glutamate, K = lysine) and non-polar (F = phenylalanine) amino acids allow it to self-assemble [13] without processes such as ultraviolet light or addition of a second chemical which can be limiting when working with live cells. Our previous work has demonstrated that extracellular matrix can be added as appropriate to the cell type; in this paper, the peptide gel is shown to form the supportive element of a model for the 3D expansion of PDX-derived cells, allowing the cells to define their own, relevant, extracellular matrix.

## Results

### Peptide gels support ex vivo culture of breast cancer PDX-derived cells

**Fig. 1:**
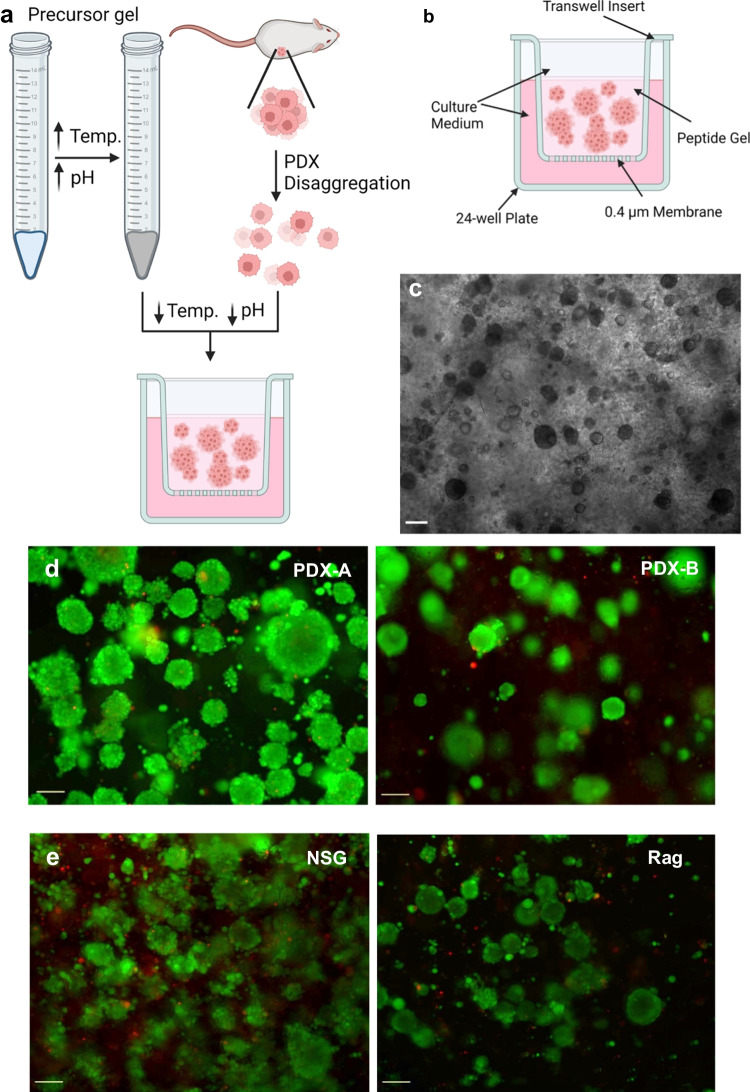
PDX-derived cells can be grown in peptide gel. **a** Diagram showing process of cell derivation, peptide gel generation and seeding of cells in peptide gel (Created with BioRender.com). **b** Diagram showing arrangement of peptide gel in transwell insert format (Created with BioRender.com). **c** Brightfield image showing PDX-A-derived cells grown in peptide gel for 8 days, scale bar shows 200 µm. **d** Live-dead staining of PDX-A- or PDX-B-derived cells grown for 8 days in peptide gel, scale bar 100 μm. **e** Live-dead staining of PDX-A-derived cells grown for 11 days in peptide gel after passage in NSG or Rag mice. Both live/dead stained (live green, dead red) with 100 μm scale bar. All gels used 10 mg/ml peptide

We initially investigated the ability of PDX-derived cells, obtained through enzymatic digestion of PDX tissue, to grow in the peptide gel. A cell-containing gel was formed by mixing a single-cell PDX-derived suspension with a precursor gel (Fig. 1a) and seeded in a transwell insert format (Fig. 1b) to maximise the availability of media to cells in the gel. In initial trials, cells from a breast cancer PDX, PDX-A, could be grown in the peptide gel, expanding to form clusters of cells from 5 days (Fig. 1c). Further experiments demonstrated that this was reproducible for PDX lines derived from different patients (PDX-B, PDX-C, PDX-D) and live/dead staining demonstrating high viability in all cases (two examples shown in Fig. 1d).

In order to confirm compatibility of the peptide gel with PDX-derived cells which had been grown in two distinct, commonly used murine TMEs, cells from PDXs which had been grown in two different mouse host strains were used. PDX lines derived from two different patients, PDX-A and PDX-B, were each grown in NOD scid gamma (NSG) or RAG2G mice (PDX-A shown in Fig. 1e), before disaggregation and seeding in the peptide gel, showing good survival and similar cluster formation in all cases.

### Breast cancer PDX-derived cells can be passaged and expanded in the peptide gel

**Fig. 2:**
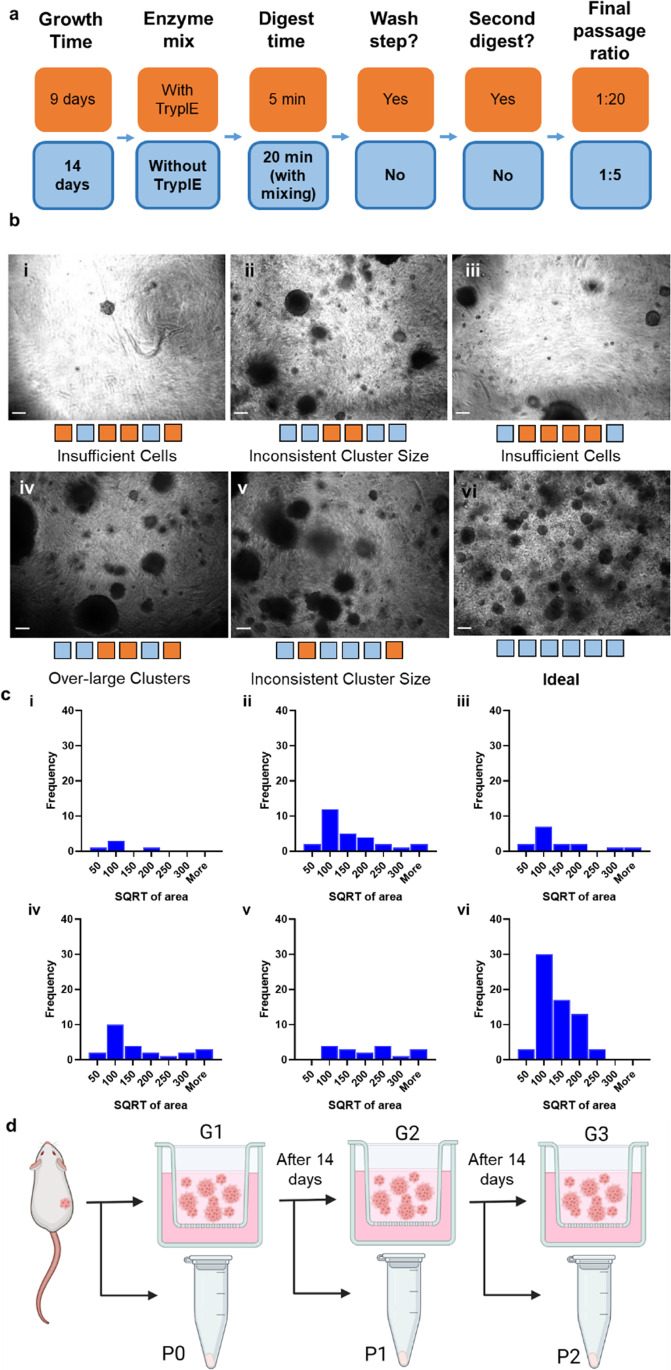
Optimisation of passage of breast cancer PDX in peptide gel. **a** Diagram of optimised elements in passage process, showing variants kept for the final process in blue (light) boxes and variants discarded as less effective in orange (dark) boxes. **b** (**i**–**vi**) Brightfield microscope images of several PDX-A-in-peptide-gel wells during optimisation of passage, with orange (dark) and blue (light) swatches below corresponding to elements in (a) representing the process used to derive them, where **(vi)** shows PDX-A cells generated using the final passage process. Scale bars 200 µm. **c** (**i**–**vi**) Histograms showing cluster sizes for each condition in (b), measured using FIJI in micrometres. **d** Diagram showing passage number nomenclature, Created with BioRender.com

Having confirmed that the peptide gel was able to support the growth of the PDX-derived cells, a protocol was developed to allow their ex vivo expansion. This was achieved by taking the cells grown in one well and transferring them into a new gel, enough to seed four wells, subsequently allowing further expansion of cell numbers. Initially, an approach analogous to that used for 2D culture was attempted. This resulted in very few cell clusters in its first iteration, and in the next attempt, specifically focused on increasing cell numbers, there were adequate clusters, but these were very inconsistent in size. With the aim of obtaining a large number of small, evenly dispersed cell clusters in the new gel, the process was optimised by attempting a new protocol at each passage, varying specific steps in the protocol (Fig. 2a). At each passage, steps in the protocol associated with less-ideal outcomes were adjusted, while those which appeared to ensure good viability were retained in subsequent passages.

We observed varying survival and evenness of cellular distribution within the well depending on the protocol used (Fig. 2b, quantified in Fig. 2c). For example, passaging a larger fraction of the cell suspension by centrifuging to remove excess medium increased the number of cell clusters in the resulting gel, while an incubation time of 20 min with pipetting to mix mid-way improved dispersal. Through the adaptation of this method, ideal variants of six parameters within the process were identified (Fig. 2a).

As a result, an optimised protocol was defined, in which cells were grown for 14 days before passage, disaggregated in the same collagenase/dispase recipe used to disaggregate the initial PDX for 20 min at 37 °C, and each well was used to seed a single new gel (effectively a passage ratio of 1:5 as each gel stock is then used to cast 5 wells). A nomenclature to describe cell samples used for downstream analysis was developed (Fig. 2d), which indicated the number of gel passages the cells had undergone and whether the analysis was done while the cells were still within the gel (G1 in the first gel, G2 in the second, etc.) or had been extracted prior to analysis (P1 after the first gel, P2 after the second, etc.).

An entirely animal-free model is ideal for patient relevance, requiring the replacement of serum-containing medium from the model. This would require an animal-free culture medium to neutralise collagenase/dispase during passage. A preliminary experiment was performed demonstrating that an entirely animal-free medium allowed passage and was supportive of growth (Fig. S1).

### The optimised protocol allows repeated passage of multiple different breast cancer PDXs with high cell viability

**Fig. 3:**
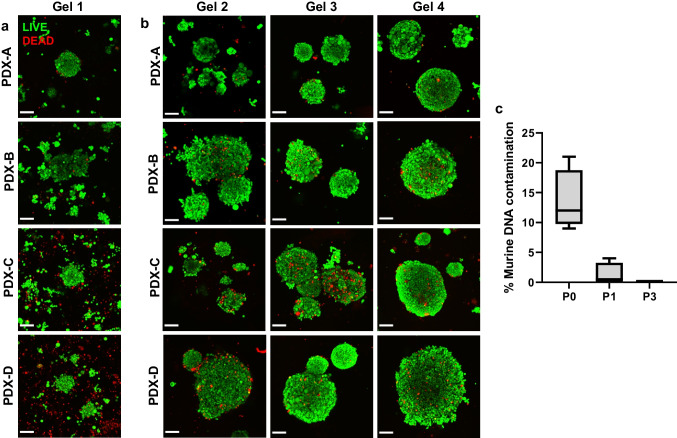
PDXs derived from different subtypes of breast cancer can be passaged with high resulting cell viability. **a** Live/dead staining (live cells stained green, dead cells stained red) of PDX-A- and PDX-B-derived cells after 8 days of growth after first seeding (G1) in 10 mg/ml peptide gel. **b** The same cultures following passage, in gel 2, gel 3, and gel 4. Scale bar 100 µm. **c** Box and whisker plot of qPCR data showing reduction of murine component to almost undetectable levels from before G1 (P0) to between G1 and G2 (P1) to between G3 and G4 (P3)

Using this optimised passage protocol, cells from four breast cancer PDXs (PDX-A and PDX-B, used for optimisation, as well as new PDXs from additional patients (PDX-C and PDX-D)) were grown over several passages to confirm broad applicability of the protocol and compare the different PDXs. In each instance, the initial (G1) passage contained relatively small clusters of live cells and some evidence of cell death (Fig. 3a), whereas in later passages (Fig. 3b), cell clusters were consistently larger than their G1 counterparts, with much less evidence of cell death. Species composition analysis by quantitative polymerase chain reaction (qPCR) using species-specific primers for murine and human DNA shows that the murine component was rapidly depleted over passage (Fig. 3c), correlating with the relative abundance of dead cells in G1. This indicates that extended culture in the peptide gels selects for human cells over the murine cell fraction. Cell viability assessment, based on late-passage (G4) samples derived from PDX-A (Fig. S2), showed continuous growth of this purified human cell population over an 8-day period.

### Immunofluorescent and histological staining can be used to assess cells grown in peptide gels

**Fig. 4:**
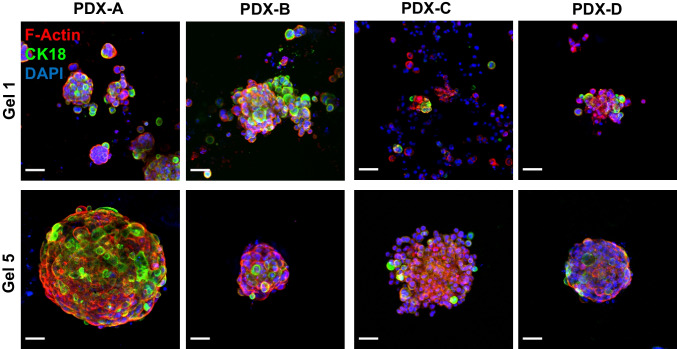
PDX-derived cells passaged in the peptide gel show F-actin (phalloidin) and cytokeratin 18 staining. Phalloidin and CK18 staining shown in red and green alongside DAPI stain for nuclei (blue) in G1 and G5 for cells derived from PDXs A, B, C and D. Scale bar 50 µm

Immunofluorescence staining for F-actin and CK18 was performed on fixed, passaged, PDX-derived cells in the peptide gels (Fig. 4). This staining demonstrated maintained CK18 expression within a subset of the PDX-derived cells cultured in the peptide gel from G1 to G5 for all four PDXs, indicating maintenance of cellular heterogeneity over extended passage. Individual cells which only show DAPI staining can also be observed in G1 samples, possibly reflecting dead, murine stromal cells.

**Fig. 5:**
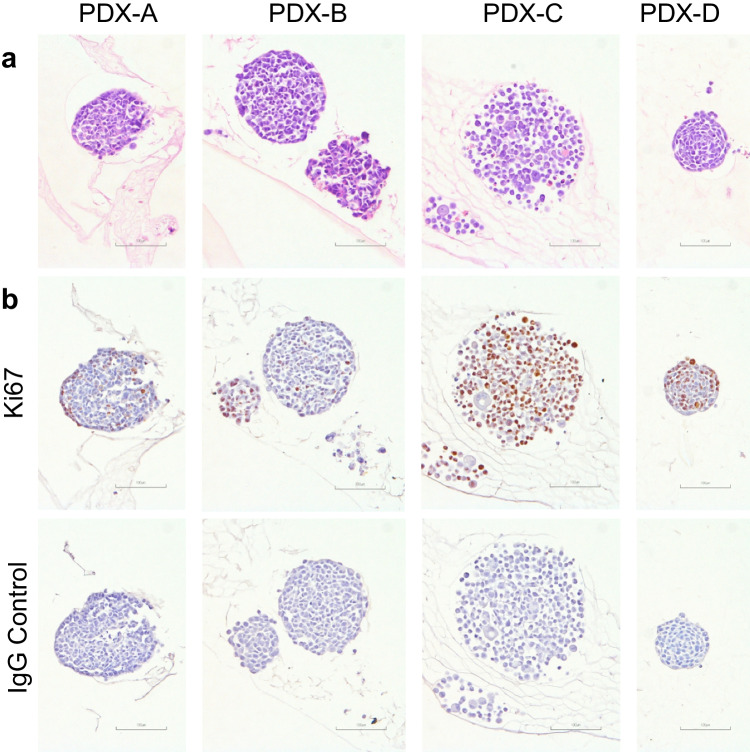
Histological sectioning and staining can be performed on PDX-derived cells in peptide gels. **a** H&E staining of 4 µm sections of cells from four PDXs grown in peptide gel to G5. **b** Ki67 with IgG control immunohistochemical staining of nearby sections of the same samples, showing unambiguous staining with varied Ki67 expression between PDXs. Scale bars 100 µm

It was also possible, by pre-embedding peptide gels in agarose blocks, to embed formalin-fixed peptide gels in paraffin and subsequently perform histological staining on sections containing cells. In addition to the classic haematoxylin and eosin staining (Fig. 5a), stains for important cancer-related proteins including Ki67 could be applied (Fig. 5b). Cell clusters derived from different PDXs show distinct cluster growth behaviours; cells derived from PDX-D appear to form in more organised layers, while the loosely packed appearance of PDX-C-derived cells in Fig. 5 is particularly distinct compared to cells derived from PDXs A, B and D. This is also evident in cell clusters observed for the corresponding PDXs when immunofluorescently stained (Fig. 4).

### Passaged cells produce matrix components, defining their own environment

**Fig. 6:**
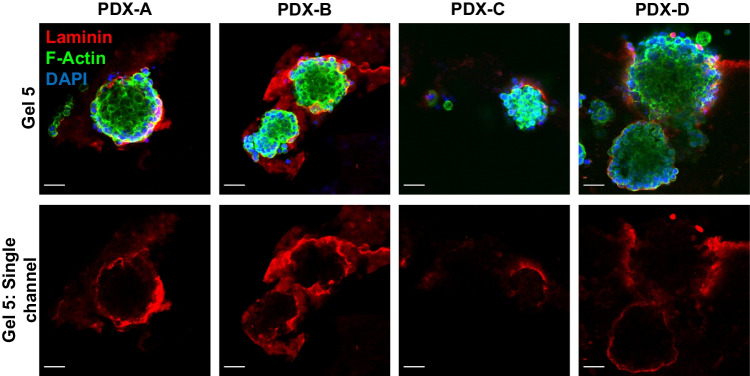
Laminin staining demonstrates the capacity of PDX-derived cells, passaged in the peptide gel, to define their microenvironment. Top: clusters of cells derived from PDX-A, PDX-B, PDX-C and PDX-D, passaged in peptide gel to gel 5 and stained for laminin (red), F-actin (phalloidin, green) and DNA (DAPI, blue). Bottom: red channel (laminin) isolated from top images, showing a “nest” of laminin for each cell cluster. Scale bar 50 µm

With the importance of the tumour microenvironment being a critical feature of a tumour, it was important to investigate whether the peptide gel model allowed the PDX-derived cells grown within it to generate their own extracellular matrix, ensuring an individualised matrix more relevant to the patient than a generic murine basement membrane extract (BME) Immunofluorescent staining of the gels for one such molecule, laminin, was performed at G5. Cells derived from all four PDXs were observed to have produced a “nest-like” laminin scaffold around their cell clusters within the peptide gel (Fig. 6), demonstrating the capacity of the cells to define their own matrix within the inert gel.

### PDX lines retain their characteristic patterns of spheroid formation after extended passage in peptide gels

**Fig. 7:**
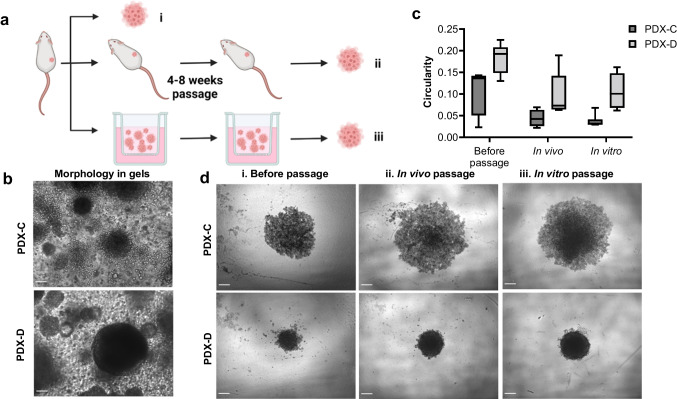
PDX lines retain characteristic spheroid formation after extended passage in peptide gels. **a** Schematic of experimental setup. **b** PDX-C and PDX-D morphology in peptide gels immediately prior to cell harvesting and spheroid formation (scale bar 100 µm). **c** Box and whisker plot showing quantification of spheroid circularity for each passage method and **d** representative images demonstrating the successful formation of spheroids with characteristic, line-specific morphology (scale bar 250 µm)

To test the hypothesis that peptide gels can replace in vivo passage as a source of patient-derived cells, a spheroid formation assay was carried out on PDX cells passaged using each method. Based on their characteristic morphologies in the peptide gel (Figs. 4 and 5), PDX-C and PDX-D were chosen for this assay. Spheroids were grown from PDX-derived cells generated from a single PDX via three different routes (Fig. 7a): directly generated from the PDX (“before passage,” Fig. 7a(i)), following 6–8 weeks additional passages in mice (“in vivo,” Fig. 7a(ii)), or following 6–8 weeks passage in the peptide gel (“in vitro,” Fig. 7a(iii)). In the peptide gels, PDX-C formed relatively loose clusters, while PDX-D clusters appeared dense with a defined, circular edge (Fig. 7b). These characteristic morphologies were also apparent in the spheroid formation assay, with PDX-C forming consistently looser spheroids than PDX-D. Circularity measurements for spheroids established using cells from PDX-C and PDX-D were in agreement regardless of passage method (Fig. 7c), with PDX-C consistently demonstrating lower circularity than PDX-D. This effect is additionally evident in brightfield images of the same spheroids (Fig. 7d), indicating that PDX-derived cells retain their characteristic phenotype after extended passage either in vivo or in vitro.

## Discussion

We have shown that close-to-patient cells, derived from PDXs, can be grown and passaged in a simple peptide gel, following the development of an optimised protocol in which key steps in the process were defined. Furthermore, viability remained high, and potentially contaminating mouse stromal cells were rapidly lost, providing a method to expand a pure population of close-to-patient human cancer cells. Importantly, immunofluorescent staining and immunohistological staining could be performed on the models, and together with assessment of spheroid formation, demonstrated that key features of the PDX-derived cells were retained, even after multiple passages in the peptide gel.

It was possible, on the first attempt, to grow PDX-derived cells from four different breast cancers in the peptide gel without supplementation beyond that provided by the same RPMI (with bovine serum and l-glutamine) used for cell line culture. Since PDXs are frequently grown in a range of different immunodeficient mouse strains, and mouse-derived stromal cells might influence the establishment and growth of cancer cells within the peptide gel, we assessed the ability of cells taken from PDXs grown in two different commonly used immunosuppressed mouse strains: RAG (RAG2-/-γc-/-) mice, which lack T- and B-lymphocytes [14] and natural killer cell function, and NSG (NOD scid gamma) mice, which have the same broad major immunodeficiencies via different mechanisms, in addition to deficiencies in innate immune components such as complement and phagocytes [15]. There was no apparent difference in the growth of cells derived from the two strains when transferred into the peptide gel, indicating the success of this method across multiple mouse strains.

Work by this group and others has previously recognised the value of ex vivo cancer models, in which PDX-derived and other close-to-patient cells can be cultured in 3D to maximise patient relevance without the limitations of in vivo models. These models often take the forms of spheroids [16, 17] or cells suspended in gels [18–21], but spheroid formation is variable depending on cell type [22], and the latter are often either formed from biologically active proteins whose effects then cannot be controlled for or require a gelation process that can alter cell behaviour such as calcium addition or UV crosslinking. The peptide gel model’s strength lies in the fact that it is inert, forming a gel under physiological conditions, and is easily customisable.

In order to expand cells further, it was necessary to identify protocols that enabled them to be passaged into new gels. We adapted standard approaches used for disaggregating tissues such as those used for establishing ex vivo experimental models from PDXs [20] and passage of 2D cultures. Parameters identified as optimal for the breast PDXs used in this study may need further modification in the future, for example, for other tumour types, but the conditions used here resulted in viable clusters of cells within the cultures, which needed to be passaged at approximately 2-week intervals. This provides a good balance between the need for frequent intervention and the potential for harvesting cells, making the cultures convenient for routine use.

Testing by qPCR confirmed that the passaged cells were almost entirely human. Five to twenty-five percent contamination by mouse cells was found in the initial disaggregated PDX tissue, consistent with descriptions in the literature of replacement of human stroma in PDXs with murine host stroma [23, 24], and yet this contamination was apparently lost on passage. This is consistent with Domenici et al.’s findings that, in contrast with 2D cultures, which retain often problematic levels of murine stromal contamination, in 3D cultures, murine stromal contamination is reduced [19]. It is possible that murine stromal contamination consists of end-stage cells which are not dividing and thus are rapidly outgrown by rapidly proliferating cancer cells.

While this does result in the loss of any stromal support provided by the murine stromal cells, it presents an opportunity to further humanise the model by the addition of human stroma [20]. Such human stromal support could be provided through direct co-culture but could also be provided indirectly, in the outer wells of the transwell setup as demonstrated previously [8]. This would avoid contamination of the PDX-derived stocks and enable downstream applications such as metabolic assays, RNAseq or drug response curves to be performed without ambiguity as to which cells are contributing to results. Alternatively, labelled human stromal cells might be added to assess the effects of direct cell–cell interactions.

Live/dead staining showed that a very large percentage of cells grown within the model were living. When performed in passage 0 compared with passage 3 or 4 gels, live/dead staining also revealed remarkably similar cell cluster structures to those at early passages, suggesting maintenance of phenotypic characteristics. As demonstrated by histology, spheroid formation and immunofluorescence, the different PDX-derived lines display individual behaviours which are maintained through passage and where relevant observed to be similar by different techniques, for example, the particularly loose packing of cell clusters derived from PDX-C.

Additionally, low levels of cancer cell death are encouraging from the point of view of potentially maintaining a greater degree of intra-tumoural heterogeneity, now understood to be present in many tumour types and a likely driver of recurrence in patients not reflected by studies in cell lines [25–27].

Most 3D cancer models are not optimised for passage; with the exception of organoids, they are used as experimental models, seeded from cell lines maintained in 2D [22] or disaggregated PDXs. Organoids, however, are routinely cultured over multiple passages to expand the cell number available for experiments [28–30]. The major limitation of organoids is the use of animal-derived basement membrane extracts in organoid culture, which introduces ill-defined, uncontrollable murine signalling molecules and reduces the 3Rs benefits of avoiding in vivo culture. The use of rat collagen instead of a whole basement membrane preparation for organoid culture [29] is a step towards a more tailored model. However, the peptide gel model provides the opportunity to take this further towards a fully human microenvironment with patient-like complexity, by enabling the creation of a 3D environment, with the potential for addition of human molecules/cells to this “blank slate” [11].

In conclusion, the peptide gel model presented here shows potential to replace in vivo passage of patient-derived xenografts, reducing the number of mice sacrificed without direct research output, allowing the patient relevance of PDX-derived cells to be increased, bringing close-to-patient 3D models to laboratories without in vivo capacity, and retaining compatibility with a wide range of commonly used assays. Having compared PDX-derived cell culture with in vivo passage, and demonstrated the model’s ability to support close-to-patient cells, a valuable next step will be to attempt culture of cells taken directly from patients.

By forming the basis of a fully tailorable cancer model with the potential to become entirely molecularly human, this method paves the way to greater uptake of 3D close-to-patient ex vivo cancer modelling and, with it, improved understanding of basic cancer science and pre-clinical model predictive capability.

## Methods

### PDX models

#### Growth of PDXs before in vitro work

Female NSG (NOD.Cg-Prkdcscid Il2rgtm1Wjl/SzJ) mice (10–14 weeks), purchased from Charles River UK and allowed to acclimatise for a week prior to use, and female Rag2G (Rag2-/- γc-/-) mice (8–10 weeks) bred in-house under PPL P375A76F, both immunodeficient strains, were used in this project. Only female mice were used as the tumours under investigation were breast lines. All mice used were of a known SPF (specific pathogen free) health status tested following FELASA testing recommendations. Mice were maintained in individually ventilated cages (IVCs) (Tecniplast, UK) within a barriered unit illuminated by fluorescent lights set to give a 12-h light–dark cycle (on 07.00, off 19.00), as recommended in the United Kingdom Home Office Animals (Scientific Procedures) Act 1986. The room was air-conditioned by a system designed to maintain an air temperature range of 21 ± 2 °C and a humidity of 55% + 10%. Mice were housed in social groups during the procedure and provided with irradiated bedding and autoclaved nesting materials and environmental enrichment (Datesand, UK). Sterile irradiated 5V5R rodent diet (IPS Ltd, UK) and irradiated water (SLS, UK) were offered ad libitum. Tissue was generated by serial passage of tumour fragments using an implant trochar (VetTech Ltd., UK) into the mammary fat pad of the mice with 50 µl Matrigel™, by licenced competent in vivo technicians. Tumours were measured weekly using Vernier callipers, and the volumes were calculated using the formula *V* = *ab*2/6, where *a* is the length and *b* is the width. Mice were also weighed weekly and given a daily health check by an experienced technician.

#### In vitro work

Phenol red-free RPMI (Sigma #R7509) + 10% Fetal Calf Serum (Sigma, #F7524) + 1% L-glutamine (Sigma, #G7513) was used as the culture medium except where otherwise stated.

#### Disaggregation of PDXs

Samples were minced using a scalpel and subsequently incubated in 12 ml collagenase/dispase solution (collagenase type II (Invitrogen 17,101–015) 100 U/ml and dispase (Invitrogen 17,105–041) 2.4 U/ml in HBSS). After 80 min of incubation at 37 °C with rotation, undigested material was separated from cell suspension; 6 ml RPMI medium was added, and the sample was washed through a 70 µm nylon mesh. Six millilitre further RPMI was used to rinse any further loose cells through the nylon mesh, and then the sample was centrifuged again, with the supernatant discarded and the cell pellet resuspended in RPMI.

### Peptide gel

#### Gel production

For each gel, 12.5 mg FEFEFKFK peptide powder (initially purchased from Cambridge Research Biochemicals and then subsequently from Pepceuticals) was added to 800 µl sterile water in a 15 ml falcon tube, vortexed for 3 min and centrifuged at 1000 rpm for 3 min before baking at 80–90 °C for at least 2 h. 0.5 M NaOH was then added incrementally to the peptide solution, vortexed to mix and briefly centrifuged. NaOH addition was repeated in 5–10 µl increments until a self-supporting, completely clear gel was formed [31]. A total of 100 µl 10 × phosphate-buffered saline (PBS) solution was added followed by vortexing and centrifugation as for NaOH, resulting in a volume of approximately 1 ml. This gel was then baked overnight at 80–90 °C. Further NaOH could be added afterwards if the gel appeared cloudy following the overnight bake. Gels were then stored at 4 °C.

#### Seeding of cells in peptide gel

Cells were disaggregated or dissociated according to the source and type and resuspended in 250 µl medium. Gels were heated at 80–90 °C for at least 2 h before seeding to ensure proper mixing, with a 37 °C incubation period immediately afterwards to ensure gel temperature would not damage cells. The 250 µl cell suspension was mixed into the 1 ml gel (bringing the peptide concentration to a final 10 mg/ml and cell number to 5 × 10^5^ cells per ml) by pipetting with care not to introduce bubbles. A total of 200 µl of the resulting cell-in-gel mixture was then pipetted into a 24-well-plate transwell insert (Greiner Bio-One 662,610) or (less often) a 96-well-plate well. Typically, 3 or 4 wells could be seeded per gel. Seeded gels were incubated at 37 °C for 10 min before addition of 1 ml of medium to each outer well and sufficient medium to fill the insert. This medium was then changed twice more in the following hour, once the following day, and every 2 to 3 days.

### Passage optimisation

Optimisation began from a passage technique analogous to the 2D passage, in which disaggregation fluid was added, incubated, then diluted in a medium to neutralise and centrifuged to remove. Six key aspects were varied during optimisation, as outlined in Fig. 2a. Growth time was the time between passages (9 days of growth or 14 days of growth), enzyme mix was the disaggregation solution composition (collagenase/dispase made up as described above with HBSS (“Without TryplE”), or with TryplE in the place of HBSS), and digest time describes how long the cells were left in disaggregation fluid before neutralisation. A “wash step” in which the cells in neutralised disaggregation fluid were centrifuged an additional time to reduce volume was necessary where large volumes of medium were used to neutralise the disaggregation fluid, owing to 1 ml of disaggregation fluid used in the outer well - by reducing the disaggregation fluid volume to just the 100 µl inside the transwell insert, this step was eliminated. An additional disaggregation step (“second digest”) following collagenase/dispase incubation, with TryplE (rather than including TryplE in the collagenase/dispase), was attempted in one case; otherwise, only a single round of disaggregation was performed. Finally, centrifuging before seeding to concentrate one well of cells into one gel (1:5 passage) was compared to diluting to 1 ml and seeding 250 µl in one gel (1:20 passage).

#### Optimised passage

Medium was removed from the insert and outer well. A total of 100 µl of disaggregation fluid was mixed into each gel by pipetting. Plates were incubated for 20 min at 37 °C, 5% CO_2_, mixing thoroughly halfway by pipetting. Insert contents were added to 1 ml medium. For the 1:20 passage, 250 µl of this mixture was seeded into a new gel; for the 1:5 passage, the medium/disaggregation fluid was centrifuged off the pellet (5 min at 300 g), and the pellet resuspended in 250 µl to seed into a new gel.

### Spheroid formation

Cells were removed from peptide gels using the optimised passage technique above or obtained from disaggregated PDX tissue using the methods given above. These were seeded into ultra-low attachment round bottom 96 well plates (Corning 7007) at 2000 cells/well and incubated for 7 days at 37 °C, 5% CO_2_. Spheroids were imaged at day 7 (see below), and spheroid circularity (4π(area/perimeter^2^)) was measured by selecting the outline of at least four spheroids per condition using the wand tool in Fiji [32].

### Growth assay

All readings were obtained using a FluoStar Omega plate reader with two additional spacers relative to standard well plates to allow for additional height due to inserts. As much medium as possible was removed from each assayed insert before adding reagents. To estimate cell viability, Prestoblue (Thermofisher, A13261) was added at 20 µl per insert directly to the top of the insert at the same concentration as for the 3D TGA model and incubated for 20 min. Inserts to be assayed again at later timepoints were rinsed three times with HBSS, returned to their co-culture plate where relevant and refed.

### Imaging

Routine fluorescent and/or live images were taken using Nikon Eclipse widefield microscope. Confocal images were taken using a Leica TCS SPE laser scanning system. Following immunohistochemical staining or haematoxylin and eosin staining, a Nikon E600 microscope was used to take colour photographs at 20 × magnification.

### Live/dead staining

Live/dead staining was performed using the Live/Dead kit from Fisher (L3224). Gels were washed twice with PBS, cut from their inserts then submerged in stain solution (2 µl ethidium homodimer and 0.5 µl calcein AM per 1 ml PBS) for 15 min in darkness at 37 °C before imaging using a widefield or confocal microscope.

### Antibody staining

Peptide gels were washed twice in PBS before fixation with 4% paraformaldehyde in PBS. The fixed gels were washed and stored in PBS, before embedding in 2% agar to allow thick sectioning using a vibratome (Leica, section thickness 500 µm).

Each gel section was covered with blocking buffer (0.5% bovine serum albumin in PBS + 0.1% triton) for 30 min prior to staining. For laminin staining, the gels were incubated overnight in a solution of primary antibody (abCam ab11575) at 1:100 dilution in blocking buffer. The gel sections were then washed in PBS and incubated overnight with secondary antibody (Invitrogen a11010) and phalloidin (F432 Thermofisher), both at 1:400 dilution. For cytokeratin 18 staining, gels were incubated overnight with 200 μl of pre-conjugated antibody at 1:100 (Thermofisher 53–9815-82) along with Alexa Fluor 633 conjugated phalloidin at 1:50 (A-22284 Invitrogen).

The gels were washed twice with PBS then incubated for 1 h in 1:1000 4′,6-diamidino-2-phenylindole (DAPI, Invitrogen D3571) before imaging.

### Q-PCR

DNA was extracted using QIAmp DNA mini kit (QIAGEN, 51,304) (with one duplicate PDX-A P0 sample additionally extracted using GenElute^tm^ genomic DNA miniprep kit (Sigma, G1N350-1KT) for comparison) from cell pellets of excess PDX cells at passage between gels or immediately after disaggregation from the PDX itself. Extracted DNA concentration was determined using nanodrop, and 50 ng DNA per well was amplified using PowerUp™ SYBR™ Green Master Mix (ThermoFisher, A25742). In a total 20 µl reaction, forward and reverse primers were included at 500 nM each. Primers were as follows (differing bases bolded):

- Human Forward: CTGATCATCCGCAAGCCTGTGAC**G**
- Human Reverse: G**C**CGGAAGGGCAGGCA**C**AT
- Mouse Forward: CTGATCATCCGCAAGCCTGTGAC**T**
- Mouse Reverse: G**A**CGGAAGGGCAGGCA**T**AT

These primers are based on those used by Ealba and Schneider [33].

Amplification was performed using ViiA 7 Real Time PCR System using the following cycle parameters: 95 °C 3 m, 40 × (95 °C 10 s, 60 °C, 30 s), 95 °C 10 s, melt curve from 60 °C through to 90 °C.

### Embedding and processing

Peptide gels were formalin-fixed for 1 h after 8 days of PDX-derived cell growth. Individual or duplicate gels were pre-embedded in blocks of 2% w/v agarose, then formalin-fixed again overnight. These were then processed overnight by immersion in 90 m 70% alcohol, 90 m 80% alcohol, 90 m 96% alcohol, 3 × (60 m 100% alcohol), 2 × (90 m 100% xylene), and 2 × (120 m paraffin). The processed agarose blocks were then embedded in paraffin and sectioned into 4 µm thick sections on polysine slides for staining.

### Immunohistochemistry

Slides were dewaxed in 3 5-min xylene baths, rehydrated in 2 1-min methanol baths and rinsed for 1 min in tap water.

For haematoxylin and eosin staining, sections were then incubated with Mayer’s haematoxylin for 3 min, rinsed for 1 min in tap water, incubated with eosin for 3 min and rinsed for 1 min with tap water before proceeding to dehydration.

To stain for Ki67, slides were then rinsed in 2 2-min PBS baths, boiled for 25 min in sodium citrate buffer (5 mM citrate and 5 mM sodium citrate, pH adjusted to 6.1), rinsed for 1 min in tap water then in 2 2-min PBS baths, blocked for 10 m in 3% peroxide diluted in PBS, rinsed in 2 2-min PBS baths, blocked for 30 m in 20% rabbit serum diluted in PBS, incubated for 60 m in 1:100 anti-Ki67 antibody (Dako M7240) or 1:217 (to match concentration) IgG control (Dako X0931), rinsed in 2 2-min PBS baths, incubated for 1 h in secondary antibody (Dako P026), rinsed in 2 2-min PBS baths, incubated for 10 m in DAB (abcam ab64238), rinsed for 1 min in tap water, incubated for 3 min in haematoxylin (Sigma-Aldrich MHS16) and rinsed for 1 min before proceeding to dehydration.

Following staining, all slides were dehydrated in 3 1-min methanol baths and 2 3-min xylene baths, then coverslipped with DPX mountant.

### Figures and statistics

Graphpad Prism was used for the generation of box and whisker plots. Cluster areas were measured using the ROI tool in ImageJ and plotted in histograms using Microsoft Excel. Other figures were prepared using ImageJ and BioRender.

### Supplementary Information

Below is the link to the electronic supplementary material.

Supplementary file1 (PDF 227 KB)

## Data Availability

Supporting data can be obtained from the corresponding author upon request.

## Abbreviations

BME: Basement membrane extract
DAPI: 4′,6-Diamidino-2-phenylindole
NSG: Nod Scid Gamma (immunodeficient mice)
PBS: Phosphate-buffered saline
PDX: Patient-derived xenograft
QPCR: Quantitative polymerase chain reaction
TME: Tumour microenvironment

## Declarations

All in vivo work was carried out under project licence PPL 3003444 and with the approval of the University of Nottingham AWERB (Animal Welfare and Ethical Review Body).

NCRI guidelines for the welfare and use of animals in cancer research, LASA good practice guidelines and FELASA working group on pain and distress guidelines were also followed, as were the ARRIVE guidelines on the reporting of in vivo experiments.

PDXs were originally established from patient samples are collected as fresh surgical material from tumour resections at UN Hospitals NHS Trust, collected with informed patient consent and National Research Ethics Service (NRES) approval (NRES REC 10/H0405/6) and used in accordance with NRES approval (NRES REC 08/H0403/37).
