# Supplementary material for: Application of a 3D hydrogel-based model to replace use of animals for passaging patient-derived xenografts
Source: In Vitro Model. 2023 May 9;2(3-4):99–111. doi: 10.1007/s44164-023-00048-x (PMC10550889; doi:10.1007/s44164-023-00048-x)
Supplement: Supplementary file 1 — Supplementary file1 (PDF 227 KB) [file 44164_2023_48_MOESM1_ESM.pdf]

Supplementary Figures for:

Application of a 3D Hydrogel-based model to replace use of animals for passaging patient-derived xenografts

Sal Jones (0000-0003-2351-8622)[1, 2], Jennifer C Ashworth (0000-0003-4189-8876)[1, 2], Marian Meakin[1], Pamela Collier[1], Catherine Probert[1], Alison A Ritchie (0000-0002-5092-4979)[1], Catherine L R Merry (0000-0002-3490-2809)[2], Anna M Grabowska (0000-0003-1507-921X)[1, 3]

[1]*Ex Vivo* Cancer Pharmacology Centre, Translational Medical Sciences, School of Medicine, Biodiscovery Institute, University of Nottingham, UK
[2]Stem Cell Glycobiology Group, Biodiscovery Institute, University of Nottingham, UK
[3]Corresponding author: anna.grabowska@nottingham.ac.uk

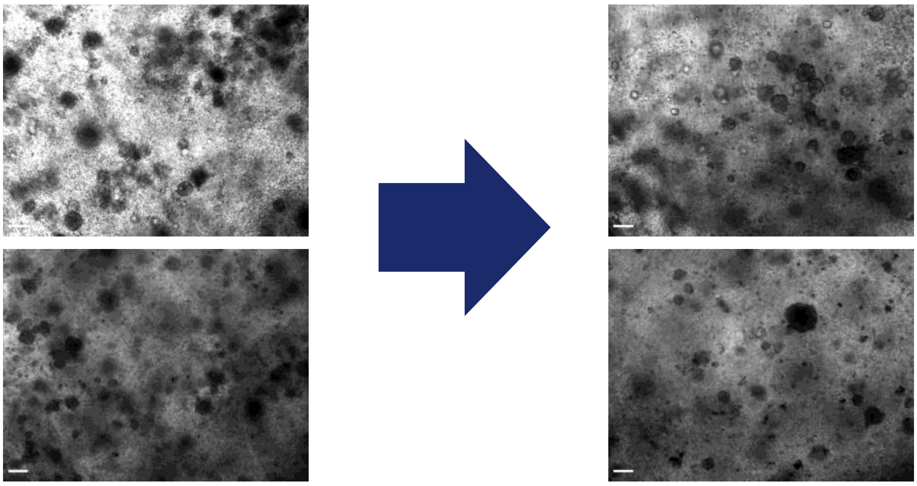

Fig. S1 *PDX-B can be grown and passaged in peptide gel using Xeno-free Tumoursphere Medium in the place of Serum-containing RPMI*

Left *brightfield images of PDX-B PDX growing in 10mg/ml peptide gel in RPMI complemented with 10% FBS and 1% L-glutamine (top) or in 10mg/ml peptide gel in Xeno-free 3D Tumoursphere Medium (XFM) (bottom), after 11 days of growth* ***Right*** *The same PDX samples after 11 days of growth following a single passage by mechanical disaggregation, with refeeds continuing to use the same medium as before passage (RPMI top, XFM bottom). All scale bars 200 µm*

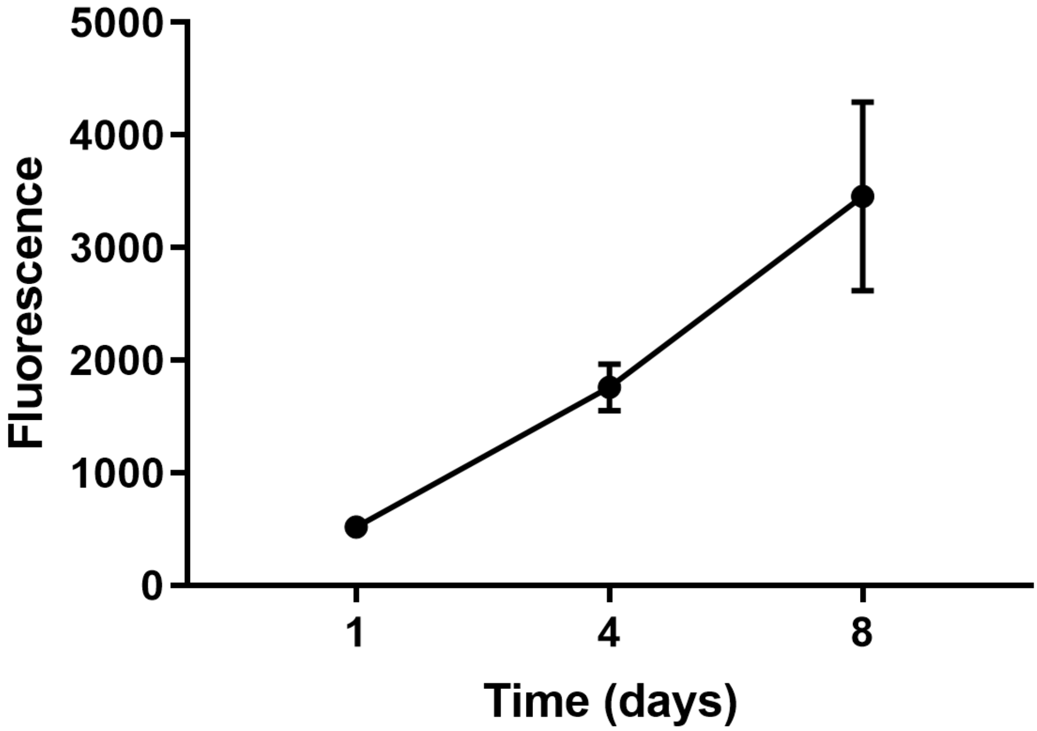

Fig. S2 *Resazurin-based metabolic assessment of proliferation/survival can be performed in the peptide gels*

Graph showing proliferation of PDX-A-derived cells at G4 based on measurements using PrestoBlue fluorescence at 24, 96 and 192 hours. n=2
